# Tipping the scales: Immunotherapeutic strategies that disrupt immunosuppression and promote immune activation

**DOI:** 10.3389/fimmu.2022.993624

**Published:** 2022-09-08

**Authors:** Ginette S. Santiago-Sánchez, James W. Hodge, Kellsye P. Fabian

**Affiliations:** Center for Immuno-Oncology, Center for Cancer Research, National Cancer Institute, National Institutes of Health, Bethesda, MD, United States

**Keywords:** immunosuppression, checkpoint blockade, immunocytokine, bintrafusp alfa, NC410, costimulatory receptors, vaccines

## Abstract

Immunotherapy has emerged as an effective therapeutic approach for several cancer types. However, only a subset of patients exhibits a durable response due in part to immunosuppressive mechanisms that allow tumor cells to evade destruction by immune cells. One of the hallmarks of immune suppression is the paucity of tumor-infiltrating lymphocytes (TILs), characterized by low numbers of effector CD4+ and CD8+ T cells in the tumor microenvironment (TME). Additionally, the proper activation and function of lymphocytes that successfully infiltrate the tumor are hampered by the lack of co-stimulatory molecules and the increase in inhibitory factors. These contribute to the imbalance of effector functions by natural killer (NK) and T cells and the immunosuppressive functions by myeloid-derived suppressor cells (MDSCs) and regulatory T cells (Tregs) in the TME, resulting in a dysfunctional anti-tumor immune response. Therefore, therapeutic regimens that elicit immune responses and reverse immune dysfunction are required to counter immune suppression in the TME and allow for the re-establishment of proper immune surveillance. Immuno-oncology (IO) agents, such as immune checkpoint blockade and TGF-β trapping molecules, have been developed to decrease or block suppressive factors to enable the activity of effector cells in the TME. Therapeutic agents that target immunosuppressive cells, either by direct lysis or altering their functions, have also been demonstrated to decrease the barrier to effective immune response. Other therapies, such as tumor antigen-specific vaccines and immunocytokines, have been shown to activate and improve the recruitment of CD4+ and CD8+ T cells to the tumor, resulting in improved T effector to Treg ratio. The preclinical data on these diverse IO agents have led to the development of ongoing phase I and II clinical trials. This review aims to provide an overview of select therapeutic strategies that tip the balance from immunosuppression to immune activity in the TME.

## Introduction

Cancer immunoediting, which is defined by its three phases, namely, elimination, equilibrium, and escape, can determine the fate of a tumor cell (1, 2). Through the *elimination phase* tumor cells are destroyed by the innate and adaptive immune system (1, 2). During the *equilibrium phase* an immune-mediated tumor dormancy can occur through several poorly understood molecular mechanisms. Lastly, tumor cells that evade the *equilibrium phase* enter the *escape phase*, by losing their immunogenicity through the effect of several immunosuppressive cell types and dysregulated signaling molecules (2). Specifically, immune-edited tumor cells going through the *escape phase* may comprise modulation in PD-L1 expression, loss of antigen presentation or decrease in several proinflammatory cytokines (2).

Hence, the main goal of cancer immunotherapy is to harness the immune system to restore immune surveillance and achieve an antitumor response. The development of immune checkpoint blockade (ICB) therapies has revolutionized the oncology field in the past two decades by providing durable clinical response in several malignant tumors (3–5). To date, the U.S. Food and Drug Administration (FDA) has approved six immune checkpoint inhibitors (ICIs): ipilimumab, which targets cytotoxic T lymphocyte antigen-4 (CTLA-4); pembrolizumab, nivolumab, and cemiplimab, which target programmed cell death-1 (PD-1); and atezolizumab, durvalumab, and avelumab, which target programmed cell death-ligand 1 (PD-L1) (4, 6, 7).

Although the percentage of patients eligible for ICI therapy has increased from 1.54% in 2011 to 43.63% in 2018 (4), the portion of patients that benefits from these therapies remains limited (8–11). For example, in metastatic colorectal cancer (mCRC) in which the 5-year survival is 15%, only 3.5%-6.5% of mCRC patients respond to ICB (9–11). In advanced cancers, such as head and neck squamous cell carcinoma (HNSCC) and advanced melanoma, only 15%-20% and 33%-44% of the patients, respectively, benefit with pembrolizumab or nivolumab (anti-PD-1) treatment (4, 8, 12–15). Patients who do not benefit from immunotherapy are known to present primary resistance, while some of the responders will relapse after a period, presenting acquired resistance (16, 17).

Several mechanisms associated with primary resistance are: 1) lack of tumor-associated proteins (i.e., low mutational burden), 2) absence of antigen presentation (i.e., deletion in beta-2-microglobulin (b2M), silenced HLA), 3) genetic T cell alterations (i.e., high oncogenic PD-L1 expression), 4) T cell desensitization (i.e., mutations in the interferon-gamma (IFN-γ) pathway signaling), 5) lack of T cells (i.e., lack of antigen-specific T cell receptors (TCRs)), 6) inhibitory immune checkpoints (i.e., VISTA, LAG-3, TIGIT, TIM-3), and 7) overpopulation of immunosuppressive cells (i.e., tumor-associated macrophages (TAMs)), and regulatory T cells (Tregs) (16). On other hand, mechanisms associated with acquired resistance include the 1) loss of T cell function (i.e., mutations in IFN-γ pathway signaling), 2) lack of T cell recognition (i.e., defects on antigen presentation), 3) escape of mutation variants (i.e., loss of tumor immunogenicity), and 4) inhibitory immune checkpoints (i.e., VISTA, LAG-3, TIM-3) (16, 18). Melanoma and Hodgkin’s lymphoma are among the cancers with an overall high response rate to anti-CTL4, and anti-PD-L1 ICIs, but with a high percentage rate of acquired resistance (18, 19).

**Figure 1:**
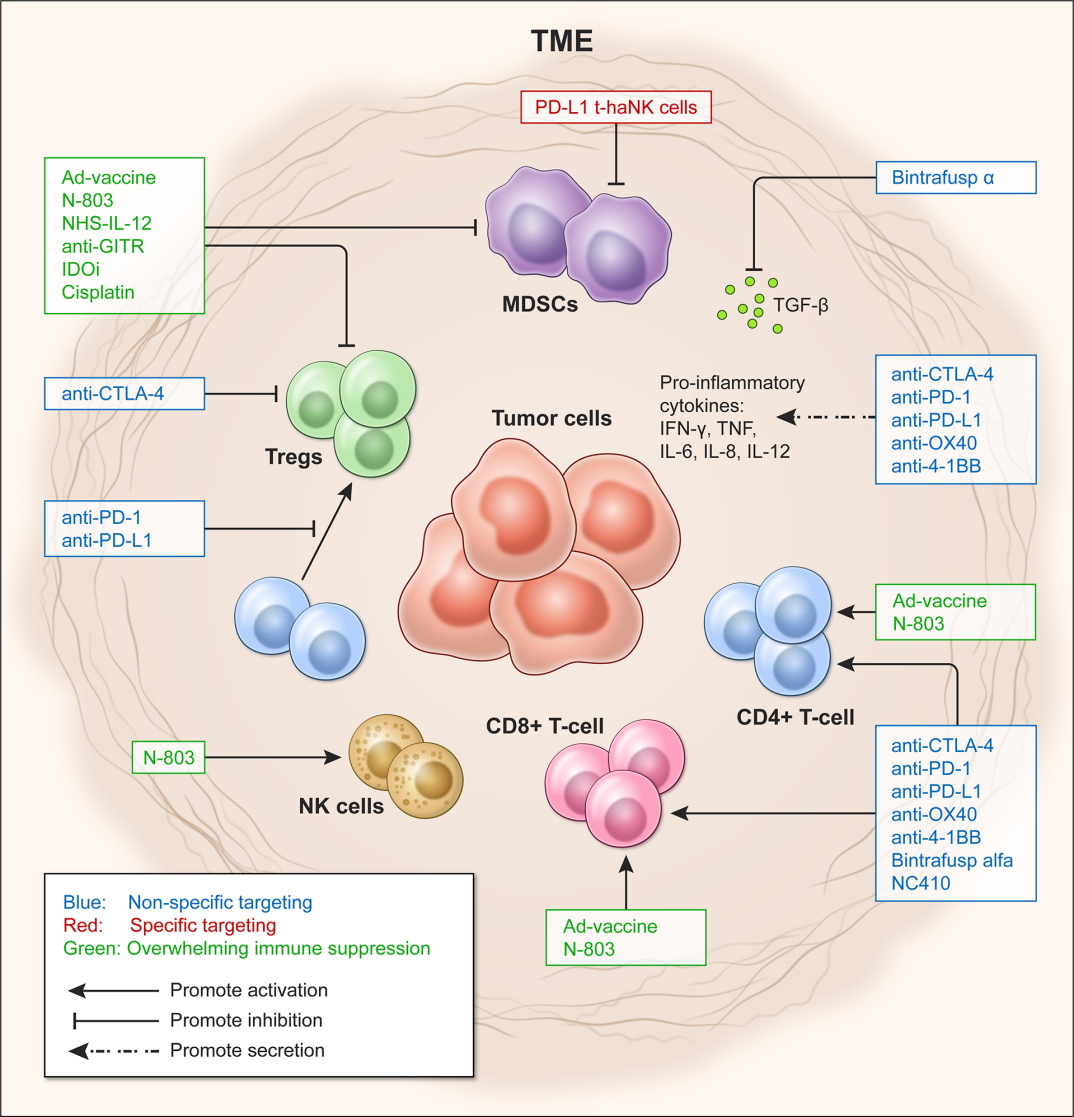
Targeting approaches to overcome immune suppression in the tumor microenvironment (TME). Effects of targeting the TME in a non-specific (blue) and specific manner (red), and by shifting Teff/Treg ratio to overcome immunosuppression (green).

These immunosuppressive mechanisms affect tumor-infiltrating lymphocytes (TILs), including helper CD4^+^ T cells, cytotoxic CD8^+^ T cells, B cells and natural killer (NK) cells (20–22), and ultimately the effectiveness of immunomodulatory strategies. Tumors with low or absent TILs, as in the case of ‘cold’ tumors, fail to respond to ICIs and are associated with poor prognosis (9, 10). Therefore, new approaches are emerging to overcome immune suppression in the TME, including ICIs in combination with costimulatory agents, metabolic modulators, and cancer vaccines, among others (16). This review discusses some of the most recent immune-oncology (IO) agents used in preclinical and clinical studies to overcome immune suppression (see **Figure 1**).

## Non-specific targeting of the TME

### Immunotherapies targeting CTLA-4 and the PD-1/PD-L1 axis

CTLA-4 and PD-1 are both checkpoint molecules expressed on T cells that upon ligand recognition hamper the cytotoxic function of effector T cells (Teff). Tumors exploit these inhibitory pathways by upregulating cognate ligands to avoid immune surveillance, thus allowing cancer cells to spread during the immunoediting *escape phase* (2, 23). Hence, the development of monoclonal antibodies (mAbs) targeting the so-called immune checkpoints has changed the landscape for patients who do not respond to conventional cancer treatments. Indeed, to date several ICIs represent the standard-of-care (SOC) for patients with advance melanoma, Merkel cell carcinoma, non-small cell lung carcinoma (NSCLC), HNSCC, MSI-CRC, and refractory Hodgkin’s lymphoma (24–27).

Ipilimumab, which targets CTLA-4, is the first-in-class FDA-approved ICI for the treatment of melanoma that does not respond to chemotherapy (6, 28). Induction of CTLA-4 signaling inhibits Teff cell activation, proliferation, and cytokine secretion. Ipilimumab and other anti-CTLA-4 mAbs block the binding of CTLA-4 on activated T cells to its ligand, B7-1 (CD80) or B7-2 (CD86), on antigen presenting cells (9), thereby impeding this inhibitory pathway (29, 30). Moreover, there is evidence that anti-CTLA-4 therapy in combination with vaccine can block immunosuppression by shifting the Teff/Treg ratio. In a poorly immunogenic melanoma mouse tumor model, combinatorial treatment with granulocyte-macrophage colony stimulating factor (GM-CSF)–transduced tumor cell vaccine and anti-CTLA-4 resulted in tumor rejection that was directly correlated with increased Teff/Treg ratio (31). In a follow-up study, it was elucidated that the activity of anti-CTLA-4 is mediated *via* selective Treg depletion within the tumor site (32). Although anti-CTLA-4 therapy has brought benefits in clinical trials of melanoma, refractory mCRC, hepatocellular carcinoma, and malignant mesothelioma, no improvement was observed in terms of overall survival (OS) in patients with metastatic castration-resistant prostate cancer (30). The mechanisms underlying the resistance to current anti-CTLA-4 therapy are poorly understood. One possible mechanism is the constitutive expression of CTLA-4 on Tregs, which can sequester the mAb from the Teff cells (24, 33). Therefore, combination therapies of anti-CTLA-4 with anti-PDL-1 and other treatment modalities represent current alternatives to circumvent immunosuppressive mechanisms present in many cancer malignancies.

Another ICI that has changed the landscape of immunotherapy in several advanced cancers are mAbs blocking the PD-1/PD-L1 axis. PD-1 (CD279), a transmembrane receptor expressed on T cells, B cells, NKs, and myeloid-derived suppressor cells (MDSCs) (34), exerts its inhibitory signaling upon binding to its cognate ligands PD-L1 (B7-H1) or PD-L2 (B7-H2), leading to a cascade of immunosuppressive mechanisms halting the cytotoxic Teff function (35–37). PD-L1 also has an essential role in converting CD4^+^ T cells into Tregs, enhancing and sustaining the expression of the transcription factor FoxP3, and maintaining the suppressive function of Tregs. Therefore, suppressing the activation of the PD-1/PD-L1 axis can partially abrogate some of these immunosuppressive mechanisms. Nivolumab and pembrolizumab, both FDA-approved anti-PD-1 mAbs, are indicated for unresectable/metastatic melanoma, and NSCLC, among other cancers well-described by Vaddepally et al. (34). Avelumab, a fully human anti-PD-L1 mAb, is approved to treat metastatic Merkel cell carcinoma, locally advanced/metastatic urothelial carcinoma and advanced renal cell carcinoma if combined with axitinib, a tyrosine kinase inhibitor (34).

In contrast to nivolumab or pembrolizumab, which are IgG4 isotype antibodies, avelumab is an IgG1 isotype mAb that can mediate antibody-dependent cell-mediated cytotoxicity (ADCC) (38). Preclinical work from Boyerinas and colleagues showed the ability of avelumab to induce ADCC on several human cancer cells including lung, breast, and bladder cancer cell lines, in the presence of peripheral blood mononuclear cells (PBMCs) or NK cells (38). Notably, the lung cancer cell line, H460, which expresses low-level MHC class and is resistant to cytotoxic T lymphocytes (CTL) lysis, was effectively lysed by purified NK cells in combination with avelumab (38). Additional work in chordoma, a rare bone cancer in the spine or skull, showed that avelumab significantly improved NK cell lysis of chordoma cells *via* ADCC *in vitro* (39). Hence, these findings demonstrate that, in addition to inhibiting the PD-1/PD-L1 axis, ADCC-mediated lysis may be another mechanism through which avelumab exerts its anti-tumor effect.

A substantial portion of patients derive limited benefit from ICI-based monotherapies. Therefore, ICIs are currently being evaluated in combination with chemotherapeutic agents, radiation, vaccines, or costimulatory molecules for those patients presenting primary or acquire resistance to ICIs (16, 23). Thus, identifying other molecules and pathways that can be targetable alone or in combination with current FDA-approved ICIs seems a feasible alternative to treat some advanced cancers.

### Landscape of immune checkpoint blockade beyond targeting CTLA-4 and the PD-1/PD-L1 axis

Additional potential targets identified for immunotherapy are the costimulatory receptors 4-1BB (also known as TNFRSF9 or CD137), OX40 (TNFRSF4, ACT35, or CD134), and glucocorticoid-induced TNFR-related protein (GITR) (TNFRS18, AITR or CD357). These molecules belong to the tumor necrosis factor receptor superfamily (TNFRSF) and have been shown to boost antitumor immune response by regulating survival, proliferation, differentiation, and effector functions of immune cells (23, 40, 41). 4-1BB receptor is expressed in activated T and B cells, monocytes, macrophages, dendritic cells (DCs), Tregs, NK, neutrophils, eosinophils, and mast cells (42). The interaction of 4-1BB with its known ligands, TNFR-associated factor (TRAF) 1 and TRAF2, on APCs triggers signals that can stimulate cell division by downregulating proapoptotic molecules, such as Bim (43). Additionally, 4-1BB receptor/ligand interaction induces the proliferation of cytotoxic T cells, the expansion of effector and memory T cells (Tm), and triggers proinflammatory T helper (Th)1 cytokine production such as interleukin (IL)-6, IL-8, IL-12, tumor necrosis factor (TNF), and INF-γ, while suppressing Th2 cytokines (IL-4, IL-5, IL-13) (44, 45).

Similarly, costimulatory receptor OX40 is expressed in activated CD4^+^ and CD8^+^ T cells, Tregs, Th cells, NK, and neutrophils (23). Immunomodulatory functions associated with OX40 interaction with its ligand, OX40L, express on APCs; these include enhancing cytokine secretion, accumulation of antigen-reactive T cells and Tm cells during the peak of the primary immune response, as well as promoting T cell proliferation by T cell receptor (TCR) antigen stimuli (46, 47). Furthermore, OX40 signaling regulates the number of CD4^+^ T cells generated during a primary clonal expansion and controls the size of the Tm pool (47, 48). Thus, OX40 is of utmost importance since quality and number of T cells are crucial to determine immunotherapy response (47). Lastly, OX40 interaction with OX40L in DCs has been shown to exert a role in DC activation, maturation (47) and promoting antitumor immunity (49).

The stimulatory checkpoint GITR is expressed in Tregs, activated NK cells, activated macrophages and DCs (50). Upon recognition of its ligand GITRL (TNFSF18), predominantly expressed by activated APCs, or with agonist antibodies, GITR signaling enhances T cell activation (50). Mechanisms associated with T cell activation by GITR include upregulation of CD25 and secretion of IL-12 and INF-γ (50). Moreover, GITR can enhance cancer vaccine activity by providing costimulatory signaling for T cell activation (51–54). Specifically, data suggest that GITR signaling shortens the threshold for CD28 signaling on CD8^+^ T cells and induces 4-1BB expression on CD8^+^ Tm (50). GITR high expression on Tregs represents a more complex mechanism, because while GITR modulation triggers Tregs expansion (50, 55), it also inhibits Tregs immune suppressive mechanisms (50, 56, 57). Indeed, growing evidence suggests that the use of anti-GITR as an agonist increases Teff/Treg ratio by increasing CD8^+^ T cell population and depleting Tregs (50, 58–60). As an example, a study using a melanoma mouse model demonstrated that costimulation of GITR with an agonist mAb achieved a loss on FoxP3 expression within the intratumoral Treg compartment (50, 60).

The therapeutic benefit of agonistic 4-1BB, OX40, and GITR costimulation has been demonstrated in several preclinical murine models of breast, colon, lymphoma, and melanoma cancers. In melanoma, 4-1BB signaling was shown to rescue chronic activated/exhausted CD8^+^ T cells (61). Importantly, when 4-1BB and OX40 agonists are used in combination with ICIs, vaccines or cytokines, a synergistic immune boost protects against poorly immunogenic cancer types. For example, a combination of costimulatory agonists anti-OX40 and anti-4-1BB mAbs with vaccine, in a breast Her-2/neu transgenic mouse model, enhanced both CD4^+^ and CD8^+^ T cell activity and proliferation associated with the retardation of tumor growth (62, 63). Combination therapy of anti-OX40, anti-4-1BB, anti-PD-L1, docetaxel, and adenovirus-based tumor antigen vaccine was shown to induce CD4^+^/CD8^+^ T cell proliferation and activity, overcome CD4^+^ and CD8^+^ T cell exhaustion, and delay tumor growth in both T cell–inflamed and non-T cell–inflamed murine tumor models (64). In terms of GITR costimulation in preclinical models, there is seminal work using agonist antibodies DTA-1 or GITRL-Fc demonstrating CD8^+^ T cell expansion and cytokine production (50). For example, a study showed tumor regression after costimulation with DTA-1 in a CT26 murine model (65). Additionally, another study showed tumor control associated with the increase in TILs and granzyme B in a Colon26 murine model [for an in-depth review, see (50)].

The clinical efficacy of several agonists for 4-1BB and OX40 in combination with ICIs is currently under investigation. Recently published results from a clinical trial (NCT02315066) of OX40 agonist, alone or in combination with a 4-1BB agonist, have shown disease control in 56% of patients associated with an increase in CD4^+^ memory T cell proliferation and activation without dose-limiting toxicities (66). In a phase I study (NCT02554812), 26.1% of the patients who received combination treatment with the 4-1BB agonist utomilumab and pembrolizumab had complete or partial responses. Furthermore, the responders had high levels of activated memory/effector peripheral blood CD8^+^ T cells (67). In an ongoing clinical trial (INTRUST), the 4-1BB agonist urelumab is being studied in advanced solid tumors including NSCLC alone or in combination with nivolumab (NCT03792724); at the time of this review, however, no results have been posted.

Recently, results were published for the first-in-human phase I clinical trial (NCT01239134) using the anti-GITR antibody, TRX518, a fully humanized Fc-dysfunctional aglycosylated IgG1K (68). During the trial, 43 patients with refractory solid tumors were treated with TRX518 monotherapy and a reduction in circulating and intratumoral Tregs was observed (68). Despite the increase in Teff/Treg ratio, however, no substantial clinical responses were observed (68). Because TRX518 monotherapy was not sufficient to activate cytolytic CD8^+^ T cells due to persistent exhaustion, the group is now evaluating TRX518 in combination with PD-1 blockade in a new clinical trial (NCT02628574) (68). A separate clinical trial (NCT02132754) is evaluating the GITR agonist MK-4166, alone or in combination with pembrolizumab in patients with advanced solid tumors (69). The combination was well-tolerated and the highest overall objective responses (ORR, 69%) were observed in ICI-naïve melanoma patients (69).

In addition to checkpoint inhibitors, tumors also produce immunosuppressive cytokines such as TGF-β and IL-8 (2). TGF-β is a pleiotropic cytokine that under physiological conditions maintains immune homeostasis and even suppresses tumor initiation (70). However, TGF-β signaling can also drive tumor progression by suppressing CD8^+^ T cells tumor infiltration (71), supporting angiogenesis (72), upregulating PD-L1 expression (73), and promoting epithelial-to-mesenchymal transition (EMT) (74). Several therapeutic agents targeting this cytokine are currently under development for the treatment of cancer.

Bintrafusp alfa, previously known as M7824, is a first-in-class bifunctional fusion protein that consists of an anti-PD-L1 antibody covalently linked to the extracellular domain of two TGF-βRII molecules that is designed to block the PD-1/PD-L1 axis while also sequestering TGF-β molecules (75, 76). Several preclinical studies have confirmed the antitumor efficacy of bintrafusp alfa and its ability to increase the immune response in triple negative breast, bladder, and HPV^+^ cervical cancer models (77, 78). In the EMT6 syngeneic breast cancer model, bintrafusp alfa resulted in an antitumor response that was associated with increased CD8^+^ T cell and NK cell activation (77). Likewise, in HTB-1 bladder, HPV^+^ SiHa cervical, and MDA-MB-231 triple negative breast cancer models in PBMC-humanized NSG mice, bintrafusp alfa achieved significant tumor growth control linked with increased tumor infiltration IFN-γ producing CD4^+^ and CD8^+^ T cells (78).

TGF-β is considered a master regulator of the EMT, and *in vitro* and *in vivo* studies have shown that bintrafusp alfa can suppress TGF-β–induced EMT in NSCLC (73). NSCLC cells treated with bintrafusp alfa showed decreased expression of the mesenchymal markers, vimentin and fibronectin, while maintaining expression of the epithelial marker E-cadherin. Likewise, a xenograft NSCLC model showed a significant reduction in vimentin expression in bintrafusp alfa-treated mice compared to untreated and anti-PD-L1-treated mice (73).

In addition to blocking PD-1/PD-L1 interaction, bintrafusp alfa can also target PD-L1 through other mechanisms. Like avelumab, the anti-PD-L1 moiety of bintrafusp alfa allows for ADCC-mediated lysis of tumor cells (39, 73). Lung, urothelial, cervical, breast and prostate cancer cells pre-treated with bintrafusp alfa showed an enhanced susceptibility to ADCC-mediated lysis by donor-derived NK cells as compared to avelumab-treated cells (73, 79, 80). Furthermore, TGF-β contributes to the upregulation of PD-L1 expression on tumor cells and TGF-β sequestration by bintrafusp alfa could subsequently result in reduced PD-L1 expression (73).

Bintrafusp alfa monotherapy, or in combination with other IO agents, is the subject of investigation in ongoing clinical trials in metastatic prostate cancer (NCT03493945), urothelial cancer (NCT04501094), colorectal cancer (NCT03436563), and HPV-associated malignancies (NCT03427411), among other cancers. A previous phase I study (NCT02517398) in advanced solid tumors showed a complete response (CR) in a patient with cervical cancer and partial responses (PR) in some patients with pancreatic and anal cancer (81). Treatment-related adverse events were observed in 4 out of 19 patients and the maximum tolerated dose (MTD) was not determined. Previous clinical trial findings using bintrafusp alfa have been well-described in other publications (75, 76).

In addition to being a physical barrier that impedes the immune cell infiltration, the tumor extracellular matrix (ECM) also regulates the activation of effectors cells (82). Collagen, which is a component of the ECM released by cancer-associated fibroblasts (CAFs), tumor cells and macrophages, has been demonstrated to impair the immune response by acting as an immune checkpoint when interacting with leukocyte-associated immunoglobulin-like receptor-1 (LAIR-1, CD305) on immune cells (83). LAIR-1 activation and signaling inhibit the function of T cells, NK cells, monocytes, and DCs (83–85). Meta-analysis of human datasets showed an association between high collagen and LAIR-1 expression with low overall survival in glioblastoma multiforme and mesothelioma and other advanced cancer types (86).

Recently, a novel fusion protein consisting of two LAIR-2 molecules—a soluble receptor that competes with LAIR-1 for binding of collagen-like domains (87)—fused to the functional IgG1 Fc tail was developed to block LAIR-1 signaling (83, 86). This molecule, called NC410, reduced human HT-29 colorectal tumor growth and promoted T cell anti-tumor activity in humanized NSG mice (86). NC410 bound to collagen-rich areas where LAIR-1^+^ immune cells were localized in the tumor (86). In the murine EMT6 breast and MC38 colon cancer models, NC410 in combination with bintrafusp alfa remodeled the tumor collagen matrix, enhanced T cell tumor infiltration and antitumor activity, and repolarized the suppressive M2 macrophages population (83). An ongoing clinical trial is evaluating the safety of NC410 in patients with advanced and metastatic solid tumors, such as ovarian, gastric, and colorectal cancer (NCT04408599). At the time of this review, no results have been posted.

## Specific targeting of immune suppressive population of the TME

### Targeting MDSCs population

Immune suppressive cells, such as MDSCs and Tregs, play a key role in promoting tumor growth by inhibiting the proliferation and cytotoxic activity of NK and T cells (2, 88). MDSCs are a heterogeneous group of immature and dysfunctional myeloid cells classified in two major subsets based on their phenotypic and morphological features: monocytic-MDSCs (M-MDSCs) and granulocytic-MDSCs (G-MDSCs) (89). MDSCs are recruited to the tumor site through signaling molecules secreted by tumor cells and tumor stroma (88, 89). Factors such as stem cell factor (SCF), GM-CSF, granulocyte colony stimulating factor (G-CSF), vascular endothelial growth factor (VEGF), and macrophage colony-stimulating factor (M-CSF) are released by tumor cells to promote the expansion of MDSC populations in the TME (89). Furthermore, the tumor induces immune suppressive functions of MDSCs by secreting inflammatory cytokines and chemokines, such as IFN-γ, IL-4, IL-6, IL-1β, and C-X-C motif chemokine ligand 1 (CXCL1) (88, 89). The main mechanisms associated with MDSC immune suppression include depriving T cells of essential amino acids and adhesion molecules (90, 91), inducing oxidative stress (90), and increasing Tregs and M2 macrophage population (90). Specifically, G-MDSCs can suppress T cell response in an antigen-specific manner, while M-MDSCs can do it using both antigen-specific and non-specific mechanisms (89, 92).

In cancer patients, tumor progression and resistance to immunotherapy are correlated with MDSC-mediated immune suppression (88, 89, 93). Indeed, MDSCs in peripheral blood of breast cancer patients are associated with advance cancer stage and metastasis (89). For example, in CRC patients, both circulating and tumor infiltrating percentages of MDSCs have shown to increase proportionally to tumor stage (89). Therefore, during the past years, preclinical and clinical studies have been focused on suppressing the MDSC population. Therapeutic agents designed to deplete the MDSC population (i.e., gemcitabine and peptibodies), block their recruitment to the tumor site (i.e., anti-CCL2 and anti-CCR5), promote their differentiation (i.e., ATRA and vitamin D3) or inhibit MDSC-mediated immunosuppression (i.e., anti-CCL2 and anti-CCR5) have been extensively reviewed in the literature (88, 89, 93). Furthermore, conventional therapies have also been reported to affect MDSC populations. For instance, clinical data have also shown a decrease of G-MDSCs population in peripheral blood of pancreatic cancer patients receiving therapy with the chemotherapeutic agent gemcitabine (94), which is the standard first-line treatment for patients with unresectable locally advanced or metastatic pancreatic cancer (95). Although several agents to deplete MDSCs are under investigation, to date no agent has been FDA approved.

Recently, a study showed that the engineered PD-L1 targeting high-affinity NK (PD-L1 t-haNK) cells may be a novel treatment that can target MDSCs (96). PD-L1 t-haNK cells were designed to express high-affinity CD16/FcγRIIIa (158V) allele, promote ADCC-mediated lysis, possess an ER-retained IL-2; circumvent the need for exogenous IL-2 in culture, and express a chimeric antigen receptor (CAR) against PD-L1 to target PD-L1 expressing cells (80, 96, 97). PD-L1 t-haNK cells were developed to target PD-L1-expressing tumor cells and were also shown to directly lyse MDSCs (96). Among the immune cells, MDSCs express high surface levels of PD-L1; however, they are not significantly targeted by NK cells in the presence of avelumab (98). *In vitro*, coincubation of PD-L1 t-haNK cells with human PBMCs from healthy donors and patients with prostate and HNSCC cancer showed a 60% reduction in peripheral MDSCs while other immune populations remained unaffected (96). *In vivo*, PD-L1 t-haNK cells trafficked in PD-L1^+^ tumors and delayed tumor growth in breast and lung cancer models in PBMC-humanized NSG mice (96).

### Targeting Tregs population

In addition to MDSCs, Tregs cells also represent a target because tumor infiltrating FoxP3^+^ CD25^+^ CD4^+^ Tregs cells are highly proliferative and suppressive (99). Tumor infiltrating Tregs express higher levels of surface molecules associated with T cell activation, such as 4-1BB, OX40, GITR, LAG-3, TIGIT, CD25, and CTLA-4 (100). Some of these molecules possess a dual role supporting Treg immune suppressive machinery. For example, Tregs use CD25 high affinity receptor to acquire endogenous IL-2 for survival while also limiting IL-2 in the TME necessary for Teff cell activation and proliferation (100, 101). CTLA-4 on Tregs interacts with CD80/86 ligands downregulating its expression in APC, resulting in inhibition of T cell activation (33, 100). Additionally, Treg cells secrete suppressive cytokine IL-10, which inhibits NK and T cells functions (102) and secrete the inhibitory molecule adenosine that suppresses Teff cell activity while maintaining a positive feedback loop for Tregs proliferation (103).

Based on the suppressive role of Tregs, many studies have been focused on the depletion or functional modulation of Tregs in the tumor milieu. Since Tregs and Teff cells share receptors, one of the main challenges of immunotherapy is depleting Tregs without depleting Teff cells. Treg-specific depleting antibody is one of the approaches used to abrogate Treg-mediated immunosuppression (100). For this purpose, surface molecules expressed at much higher levels on Tregs than T cells are used as targets, such as CD25, CTLA-4, GITR, 4-1BB, OX-40, LAG3, TIGIT, CCR4, and CCR8; tumor burden control has also been observed (58, 100, 104, 105). For instance, in a murine pancreatic tumor model, when an anti-CD25 antibody to deplete Tregs was combined with vaccine, mice showed smaller tumors, longer survival, and a tumor-specific immune response (106). In addition to the commonly used systemic administration of Treg-specific antibodies, local delivery to the tumor site can be performed. A recent study conjugated anti-CD25 mAb with photoactivatable dye to selectively damage the cell membrane of Tregs upon near-infrared (NIR) light exposure, resulting in the tumor regression of the Lewis lung carcinoma model (100, 107). The efficacy of CD25-depleting antibodies to promote antitumor immunity is still unclear. While anti-CD25 depleting antibodies can decrease Treg populations, they can also target activated Teff cells that also express CD25 (108).

Further studies have interrogated Treg depletion in cancer immunotherapy using the agonistic anti-GITR or small molecule drugs in low doses, such as the alkylating agent cyclophosphamide (99). Tyrosine kinase inhibitors (TKIs) are also used to achieve Treg depletion and augment antitumor immunity (109). For example, a study using the TKI, imatinib, to treat chronic myelogenous leukemia (CML) patients, observed a depletion of Tregs and a significant increase in effector/memory CD8^+^ T cells in CML patients in complete molecular remission (CMR) compared to non-CMR patients (109).

CCR4, a chemokine receptor predominantly expressed on Tregs (110), has been investigated as a target for Treg depletion and several clinical trials using a humanized anti-CCR4 IgG1 mAb with a defucosylated Fc region, known as mogamulizumab (KW-0761), are underway. In a phase Ia study, KW-0761 was shown to efficiently deplete FoxP3^+^ Tregs cells with no toxicity in lung and esophageal cancer patients (111). However, the treatment also showed a limited reduction in Th1 CD4 T cells and CD8 T cells and a significant reduction in Th2 and Th17 CD4 T cell populations (111). Currently, KW-0761 in combination with chemotherapy agents or with ICIs continue under evaluation for several advanced solid tumors.

## Overwhelming immune suppression

### Shifting Teff/Treg ratio through chemotherapy

As mentioned earlier, Tregs in the TME can decrease the number of cytotoxic Teff cells, block Teff cell activation, and maintain a positive feedback loop for Treg accumulation. In fact, a high Teff to Treg (Teff/Treg) ratio in murine models is associated with response to ICIs, while a low Teff/Treg ratio is associated with ICI treatment resistance (16). In the clinic, a low Teff/Treg ratio also correlated with poor prognosis in patients with melanoma (112), breast (113), ovarian (114), and gastric cancers (115). Conversely, a high CD8^+^ TILs/Treg ratio in patients with epithelial ovarian cancer was associated with better prognosis (114). Therefore, efforts in improving immunotherapy outcomes have been focused on increasing the Teff/Treg ratio in the TME.

Clinical and preclinical data using taxanes, antimetabolites, and DNA-alkylating drugs as monotherapies or in combination with IO agents have shown to increase Teff cells and decrease Tregs in several cancer models (21, 116–118). For example, a study using cisplatin, a platinum-based chemotherapy, in combination with vinorelbine, a tubulin inhibitor-based chemotherapy, showed a sustained depletion in the number of Tregs with an increase in CD4^+^ Teff cells in a murine lung adenocarcinoma model (117). Here, a 1.5 and 2-fold increase in CD4^+^ Teff/Treg ratio, 4 and 7 days after a cisplatin/vinorelbine chemotherapy regimen, respectively, was observed (117).

In another study, clinical data have shown that patients sensitive to cisplatin-based neoadjuvant chemotherapy (119) exhibit a 5-year survival rate of 80-90%, while patients resistant to the therapy exhibit a 5-year survival rate of 30-40% (118). To interrogate the difference between responders and non-responders to NAC therapy, a study analyzed tumor biopsies from a cohort of muscle invasive bladder cancer patients and found that, individually, neither CD8^+^ T cell nor Treg density was associated with NAC response but NAC response was strongly associated with CD8^+^ T cell/Treg ratio (118). However, these findings are not representative of all cancers, as indicated in another study that showed no correlation between the CD8^+^ T cells/FoxP3^+^Treg ratio and response to therapy in HNSCC patients treated with a chemotherapy regimen (116). These contradicting observations raise the question whether Teff/Treg ratio is a good indicator of the immune response to chemotherapy.

A body of data has set the rationale for the development of clinical trials testing ICIs in combination with chemotherapy agents in cancers such as NSCLC (21, 120). For example, although nivolumab outperformed platinum-based chemotherapy for the treatment of NSCLC patients, chemotherapy may synergize with nivolumab through immunogenic modulation and abrogation of immunosuppressive cell populations (120, 121). An ongoing phase 1/2 clinical trial is evaluating the safety and efficacy of nivolumab and ipilimumab in combination with immunogenic chemotherapy for patients with advanced NSCLC (NCT04043195). Additionally, a phase 3 clinical trial (IMpower130) showed a significant improvement in OS and progression-free survival in stage IV NSCLC patients who received atezolizumab (anti-PD-L1 mAb), in combination with chemotherapy (carboplatin plus Nab-paclitaxel) compared to patients receiving standard-of-care chemotherapy alone (21).

### Shifting Teff/Treg ratio through vaccines and immunocytokines

Cancer vaccines engage the antitumor immune response to generate tumor-specific effector cells (122). A cancer vaccine has four key components, the transgene of a tumor-specific antigen (TSA) or a tumor-associated antigen (TAA), the formulation, an immune adjuvant, and the delivery vehicle (123). After vaccine administration, the professional APC (i.e., DCs) processes the antigen, presents it on its surface *via* MHC molecules, and induces a polyclonal CD4^+^ and CD8^+^ T cell response (124–126). In preclinical studies, cancer vaccines have been shown to inhibit tumor growth and promote TILs while decreasing FoxP3^+^ Tregs, thereby improving the Teff/Treg ratio (119, 126–128). In the clinic, cancer vaccines have been proven safe; however, they lack clinical efficacy as monotherapy (122). This treatment modality, nevertheless, represents a feasible backbone for combination therapy, wherein other immune-oncology agents can capitalize on the tumor antigen-specific immune cells elicited by the vaccine.

Studies to test the efficacy of vaccines in combination with immunocytokines, which are antibody-cytokine fusion proteins (129), to treat tumors and to circumvent immunosuppressive mechanisms are underway. For instance, a preclinical study using the adenovirus-based vaccine targeting the carcinoembryonic antigen Ad-CEA, which is an oncofetal tumor antigen, in combination with N-803, an IL-15 superagonist complex consisting of an IL-15 mutant (IL-15N72D) bound to an IL-15 receptor α/IgG1 Fc fusion protein (130–132), showed improved immune response and antitumor activity in a CEA-expressing MC38 murine colon carcinoma model (51). Ad-CEA + N-803 combination therapy resulted in increased CEA-specific CD8^+^ T cells in the periphery compared to treatment with Ad-CEA or N-803 alone. This suggests that the expansion of CEA-specific T cells may be due to the inflammatory stimulus of N-803 (51, 131), in concordance with an earlier study showing the positive effect of N803 on NK and CD8+ T cell populations (133). Similarly, the Ad-CEA + N-803 combination also resulted in decreased CD4^+^CD25^+^FoxP3^+^ Treg population, effectively increasing the Teff/Treg ratio when compared to Ad-CEA or N-803 monotherapies (51). Currently, there are several clinical trials evaluating the safety and efficacy of Ad-CEA + N803 in combination with standard of care and other immune-oncology agents (NCT04247282, NCT03387085, NCT03387111, and NCT03563157); results have yet to be posted.

Another immunocytokine that is currently being studied in combination with cancer vaccines is NHS-IL12, an engineered immunocytokine composed of two molecules of IL-12 fused to a tumor necrosis-targeting human IgG (NHS76) (134). The combination of MUC1-targeting vaccine and NHS-IL12 delayed the growth of MUC1-expressing tumors and promoted a robust peptide-specific CD4+ T cell proliferation (135). NHS-IL12 has also been shown to cause a shift from an immunosuppressive to inflammatory TME by promoting the activation of CD4^+^ and CD8^+^ T cells, increasing the CD4^+^/CD8^+^ T cells to MDSC ratio, and reducing intratumoral TGF-β (136). In another preclinical study, a human papillomavirus (HPV) therapeutic vaccine in combination with NHS-IL12 controlled the tumor growth of an HPV^+^ murine tumor, which was associated with the expansion of activated CD8+ T cell population in the TME (137). Treatment efficacy was further enhanced when HPV vaccine + NHS-IL12 was combined with bintrafusp alfa. A phase I/II trial evaluating the safety, overall response rate, and survival with the HPV vaccine + NHS-IL12 + bintrafusp alfa combination in patients with advanced HPV-associated malignancies is currently underway (NCT04287868).

The outbreak of COVID-19 in 2020 not only boosted the messenger RNA (mRNA) technologies for the development of SARS-CoV-2 vaccine but also renewed interest in mRNA vaccines as an alternative treatment strategy for cancer (124). In fact, over twenty mRNA-based immunotherapies have entered clinical trials for the treatment of solid tumors, including NSCLC, advanced melanoma, CRC, pancreatic and bladder cancers, and metastatic CEA-expressing solid tumors (126). Currently, several RNA types are under investigation for cancer vaccines, including virus-derived self-amplifying (49) RNA, non-replicating unmodified mRNA and modified mRNA (126). SAM-RNA vaccines, which encode for tumor antigen(s) as well as genes for viral RNA replication machinery, have been shown to induce higher antigen expression and elicit a stronger immune response compared to other mRNA type vaccines (138, 139). SAM-RNA vaccines can be delivered in the form of plasmid DNA, *in vitro* transcribed (IVT) RNA, and virus-like RNA particles (138).

An alphavirus SAM-RNA vaccine, known as virus-like replicon particle (VRP)-CEA (6D) vaccine or AVX701, has been investigated in two clinical trials for the treatment of stage III CRC and advanced or metastatic CEA-expressing tumors (NCT00529984, NCT01890213) (126, 140). The components of this platform have been designed to improve vaccine efficacy – the VRP promotes tropism towards DCs while CEA (6D), which has an Asn to Asp substitution in position 6, enhances recognition by cognate CD8^+^ T cell receptor (140). Crosby et al. reported that among the stage IV cancer patients treated with VRP-CEA (6D) vaccine, the median follow-up was 10.9 years and the 5-year relapse-free survival (RFS) was 17% (140). Among the stage III cancer patients, the survival at a median follow-up of 5.8 years was 100% and the 5-year RFS was 75%. Patients in the stage III cancer cohort were shown to have increased CEA-specific CD8^+^ Teff cells and decreased FoxP3^+^ Tregs (140). The shift in the Teff/Treg ratio after VRP-CEA (6D) vaccination suggests an effective immune modulation and provides a rationale for the combination of this virus-like SAM-RNA vaccine with ICB (140). Other mRNA vaccines using different formulations as delivery systems and for the treatment of other malignancies are currently under study; they are well described by others (125, 126, 141, 142) and beyond the scope of this review.

### Shifting Teff/Treg ratio through inhibition of immunosuppressive pathways

Another approach to promote immune response is to inhibit the immunosuppressive molecule indoleamine 2,3-dioxygenase (143) (51, 64, 144). IDO secretion promotes apoptosis of Teff and the activation of Tregs mainly by reducing the availability of the amino acid tryptophan and increasing its metabolite, kynurenine, in the TME (23, 145). The immunosuppressive effect fostered by IDO is also magnified in the TME, since IDO is induced by several pro-inflammatory signals (IFN-γ, TNF-α, TGF-β), resulting in its expression by tumor, immune, and stromal cells (23, 145). A preclinical study investigating the effect of the IDO inhibitor (IDOi) epacadostat in combination with Ad-CEA, N-803, OX40 agonist, and GITR agonist demonstrated antitumor efficacy in a MC38-CEA murine tumor model that was associated with an expansion of splenic and tumor infiltrating CD8^+^ T cells (51). Furthermore, not only did the combination promote the expansion of Teff cells over Tregs, but it also dampened the suppressive activity of Tregs (51). Additionally, analysis of serum from mice treated with the combination therapy showed significant reduction in kynurenine levels compared to control.

IDO inhibitors are currently being evaluated in combination with checkpoint inhibitors (22). Several clinical trials are evaluating how blocking the enzymatic activity of IDO inhibits the suppressive mechanisms fostered by IDO in the TME (146). However, a recently concluded phase 3 clinical trial, ECHO-301, evaluating epacadostat in combination with pembrolizumab, failed to show any clinical benefit in unresectable or metastatic melanoma patients (143). Despite these findings, rather than discard the idea that blocking IDO pathway will improve the immune response, researchers should rethink which IO agents should be combined with IDOi as well as optimal dosages. As described by Fabian et al., the combination of a specific antigen vaccine and costimulating agents with the IDOi epacadostat showed a robust antitumor activity and an immune response (51). Other clinical trials are also evaluating costimulation through anti-GITR agonist alone in solid tumors (NCT 01239134), or in combination with an IDOi and checkpoint inhibitors in patients with glioblastoma (NCT03707457).

## Future perspectives

Immunosuppression is a hurdle to overcome for the success of immunotherapeutic strategies in cancer treatment (147). However, not only should immunosuppressive mechanisms be addressed, but immunomodulatory mechanisms promoting T cell priming and activation should also be met. This often requires a treatment strategy that combines different agents to target different facets of the tumor-immunity interactions. Combination therapies, however, also come with their own challenges. For such strategies, it is crucial to interrogate not only their antitumor efficacy, but also the safest doses that maintain effectiveness, as well as the schedules for the agent’s administration. Moreover, a deeper understanding of the known immunosuppressive pathways, as well as identifying new ones, could enable the development of immunotherapies relevant to many cancers.

## Author contributions

All authors listed have made a substantial, direct, and intellectual contribution to the work, and approved it for publication.

## Funding

This research was supported by the Intramural Research Program of the Center for Cancer Research, National Cancer Institute, National Institutes of Health (NIH).

## Acknowledgments

The authors thank Debra Weingarten for her editorial assistance in the preparation of this manuscript.

## Conflict of interest

The authors declare that the research was conducted in the absence of any commercial or financial relationships that could be construed as a potential conflict of interest.

## Publisher’s note

All claims expressed in this article are solely those of the authors and do not necessarily represent those of their affiliated organizations, or those of the publisher, the editors and the reviewers. Any product that may be evaluated in this article, or claim that may be made by its manufacturer, is not guaranteed or endorsed by the publisher.
